# Molecular Study of *Theileria annulata* and *Anaplasma* spp. in Ixodid Ticks from Southern Regions of the Republic of Kazakhstan

**DOI:** 10.3390/vetsci12090901

**Published:** 2025-09-17

**Authors:** Zaure Z. Sayakova, Saltanat A. Kenessary, Ainur A. Zhaksylykova, Bagzhan M. Abdimalik, Eleonora A. Kydyrkhanova, Dinara K. Kamalova, Anara Ryskeldina, Yekaterina O. Ostapchuk, Christine M. Budke, Aida M. Abdybekova

**Affiliations:** 1 Kazakh Scientific Research Veterinary Institute LLP, Almaty 050016, Kazakhstan; zzsayakova@mail.ru (Z.Z.S.); kenesary0200@mail.ru (S.A.K.); ainusik_jan_91@mail.ru (A.A.Z.); bagjanab9@gmail.com (B.M.A.); aryssova2.8.1@gmail.com (E.A.K.); 2 National Center for Biotechnology LLP, Astana 010000, Kazakhstan; diwr@mail.ru (D.K.K.); anararyskeldina@gmail.com (A.R.); 3 Almaty Branch of the National Center for Biotechnology, Almaty 050054, Kazakhstan; katyostapchuk@gmail.com; 4 College of Veterinary Medicine & Biomedical Sciences, Texas A&M University, College Station, TX 77843, USA; cbudke@cvm.tamu.edu

**Keywords:** *Theileria*, *Anaplasma*, ixodid ticks, cattle, PCR, sequencing, southern region of Kazakhstan

## Abstract

Ixodid ticks are vectors of pathogens that cause dangerous infectious and parasitic diseases in animals, leading to reduced productivity and, in some cases, mass mortality. In Kazakhstan, information on tick fauna and their epizootological role in the transmission of hemoparasites is limited. This study aimed to determine the species composition of ixodid ticks parasitizing cattle in the Almaty, Zhambyl, and Turkistan oblasts, and to assess their potential role in the transmission of *Theileria* and *Anaplasma* spp. A total of 3121 ixodid ticks were collected from cattle, belonging to the genera *Hyalomma* (86.9%; 2711/3121), *Rhipicephalus* (8.5%; 266/3121), *Dermacentor* (3.2%; 101/3121), and *Haemaphysalis* (1.4%; 43/3121). Morphological identification revealed that ticks of the genus *Hyalomma*, including *Hyalomma scupense* (31.7%), *Hyalomma asiaticum* (27.9%), and *Hyalomma anatolicum* (19.6%), were the predominant species. *Rhipicephalus annulatus* was recorded for the first time in the Almaty and Zhambyl oblasts. Partial sequencing of the *cox1* gene confirmed the species identification obtained by morphological examination. A total of 113 representative ticks were subjected to DNA extraction for the identification of *Theileria* and *Anaplasma* species using conventional PCR with primers targeting the *28SrRNA* and *GroEL* genes, respectively. No *Anaplasma* spp. were detected in the analyzed tick samples. *Theileria annulata* DNA was identified in seven nymphs of *Hy. scupense* (6.1%) collected in the Zhambyl oblast, and for the first time in Kazakhstan, in one female *Rhipicephalus annulatus* (0.9%) collected in the Almaty oblast. The overall infection prevalence of *Theileria annulata* was 7.0% (8/113).

## Simple Summary

Ticks are small parasites that feed on the blood of animals and can spread serious diseases to livestock, reducing milk and meat production and sometimes causing high death rates. In Kazakhstan, there is little information about which tick species infest cattle and which can carry harmful blood parasites. This study was conducted in three regions of southern Kazakhstan (Almaty, Zhambyl, and Turkistan) to identify the tick species found on cattle and check if they carry *Theileria* or *Anaplasma*, two important disease-causing microorganisms. A total of 3121 ticks were collected, most belonging to the genus *Hyalomma*. The most common were *Hyalomma scupense*, *Hyalomma asiaticum*, and *Hyalomma anatolicum*. For the first time, the species *Rhipicephalus annulatus* was found in Almaty and Zhambyl. Molecular analysis showed no *Anaplasma* spp. However, *Theileria annulata*, the parasite that causes theileriosis in cattle, was found in several *Hyalomma scupense* ticks from Zhambyl oblast and, for the first time in Kazakhstan, in one *Rhipicephalus annulatus* tick from Almaty oblast. These results improve knowledge of tick species in Kazakhstan and show the importance of continued monitoring to protect animal health and farming.

## 1. Introduction

Currently, the global fauna of ixodid ticks (family *Ixodidae*) encompasses 729 species [1]. In the regions bordering Kazakhstan, the following levels of species diversity have been reported: 68 species in the Russian Federation [2], 42 in Kyrgyzstan [3], 33 in Uzbekistan [4], 39 in Turkmenistan [5], and 111 in China [6].

Within Kazakhstan, approximately 42 species of ixodid ticks have been recorded, representing five genera: *Dermacentor*, *Haemaphysalis*, *Hyalomma*, *Ixodes*, and *Rhipicephalus.* In addition, occasional records exist for the European species *Ixodes ricinus* [7] and the African species *Hyalomma rufipes* [8], which are most likely introduced by migratory birds [9,10]. The majority of tick species in Kazakhstan exhibit wide distribution ranges and are classified as pasture-questing ectoparasites.

In the southern regions of the country, 18 ixodid tick species have been identified, 12 of which feed on domestic animals, including cattle [11,12].

Among the pasture tick species of the genus *Dermacentor* common in southern Kazakhstan, the following have been recorded: *D. marginatus*, *D. niveus*, *D. reticulatus*, and *D. pavlovskyi.* These species have a limited distribution in the southern regions and can use cattle as feeding hosts [13,14]. Representatives of the genus *Dermacentor* play an important role in epizootiology, serving as vectors of various infectious diseases of domestic animals [15,16].

The genus *Haemaphysalis* is represented in southern Kazakhstan by three species: *Ha. erinacei*, *Ha. punctata*, and *Ha. sulcata. Ha. erinacei* is distributed in desert and semi-desert zones; it seldom infests domestic animals, preferring instead small mammals and occupying primarily rodent burrows. By contrast, the pasture species *Ha. punctata* and *Ha. sulcata* have a more localized distribution but are capable of parasitizing livestock. In the neighboring Kyrgyz Republic, *Anaplasma bovis* and *Anaplasma capra* were isolated from *Ha. punctata* [17].

Ticks of the genus *Rhipicephalus* are widespread throughout southern Kazakhstan and are generally associated with pasture habitats. An exception is *Rhipicephalus schulzei*, which is closely linked to the burrows of ground squirrels and, less frequently, other rodent hosts. The species *Rh. turanicus*, *Rh. pumilio*, *Rh. rossicus*, and *Rh. annulatus* readily parasitize domestic animals, including cattle. In Kyrgyzstan, *Rh. turanicus* and *Rh. annulatus* have been identified as vectors of *Anaplasma bovis* and *A. ovis* [17], while in China, *Rh. turanicus* has also been found to harbor *Anaplasma ovis* and *Theileria ovis* [18].

Among the representatives of the genus *Ixodes* occurring in southern Kazakhstan, the majority are burrow-dwelling species, primarily *I. occultus* and *I. crenulatus*. However, at present there are no reliable data confirming the role of the genus Ixodes in the transmission of theileriosis.

Ticks of the genus *Hyalomma* occupy a leading ecological niche in the arid and semi-arid zones of Kazakhstan. Under these conditions, they often dominate numerically among the ectoparasites infesting cattle [12]. Within this group, *Hyalomma scupense*, *Hy. marginatum*, *Hy. asiaticum*, and *Hy. anatolicum* are considered the principal vectors of *Theileria* spp. [19,20]. Other genera—*Rhipicephalus*, *Dermacentor*, and *Haemaphysalis*—also play a significant role in the epidemiology of tick-borne infections in Central Asia [21,22].

In Kazakhstan, where livestock production constitutes one of the leading sectors of agriculture, hemoparasitic diseases of cattle are reported annually, sometimes resulting in substantial mortality. In the southern regions, theileriosis is registered predominantly during the spring–summer season and frequently occurs as mixed infections with anaplasmosis and babesiosis. The disease, which most often manifests in an acute form, is characterized by severe systemic disturbances, progressive emaciation, and a high probability of fatal outcomes [23,24,25,26,27].

Molecular studies conducted in various regions of Kazakhstan have identified the DNA of blood-parasitic pathogens, including *Theileria annulata*, *Babesia caballi*, *Anaplasma phagocytophilum*, *Babesia occultans*, *Theileria ovis*, *Theileria orientalis*, *Theileria equi*, and *Anaplasma ovis*, in four ixodid tick species—*Dermacentor marginatus*, *Hyalomma asiaticum*, *Hyalomma scupense*, and *Hyalomma anatolicum* [28,29].

At the same time, according to the available literature, there are no data on the molecular detection of hemoparasites in ticks at different developmental stages (egg, larva, nymph, adult), which represents a significant gap in epizootiological research.

Given the specific climatic and ecological conditions of southern Kazakhstan—which promote both high population densities and active dispersal of ixodid ticks—comprehensive studies of their bioecology, seasonal dynamics, geographical distribution, and role in pathogen transmission are of critical importance. Such investigations allow the identification of the most epidemiologically relevant species circulating in the region and facilitate the assessment of associated epizootic risks.

Efforts to control theileriosis and other vector-borne parasitic diseases in Kazakhstan are hindered by the absence of effective preventive measures and the high density of domestic animal populations, which together create favorable conditions for the proliferation of hematophagous arthropods. Despite the considerable veterinary significance of this issue, detailed studies on the distribution, species diversity, seasonal activity, and epidemiological importance of ixodid ticks in the country remain insufficient.

The present study provides essential data on tick species composition, seasonal activity, and their involvement in pathogen transmission in one of the major livestock-producing regions of Central Asia. The combined application of morphological and molecular identification methods highlights the value of integrative taxonomic approaches and underscores the persistent threat posed by *T. annulata* to cattle health in the region.

## 2. Materials and Methods

### 2.1. Ethical Approval

The study was approved by the Local Ethical Committee of the «Kazakh Scientific Research Veterinary Institute» LLP, Almaty, Kazakhstan (Approval 14 November 2022). The conclusion of the ethics commission was issued when submitting the project to the competition. Animals were treated with humane care in accordance with ethical guidelines for animal research.

### 2.2. Tick Collection and Identification

**Figure 1:**
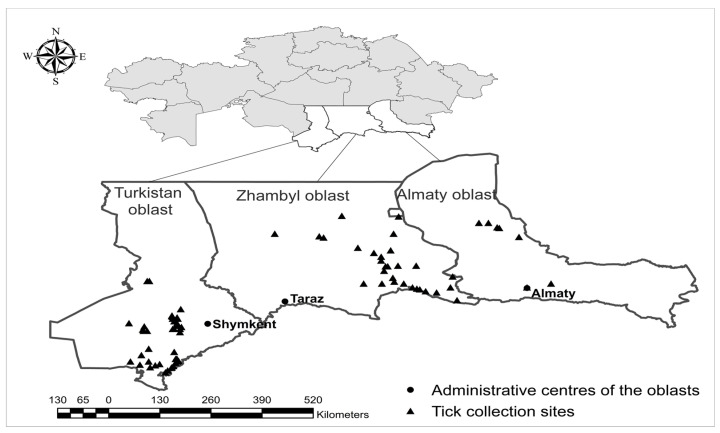
The administrative centers and administrative units (oblasts) of the southern region of Kazakhstan are shown. The triangles indicate the areas where ticks were collected.

Assessment of tick infestation and tick collection was conducted on cattle (*Bos taurus*) at private farms in the Almaty, Zhambyl, and Turkistan oblasts from March to November 2024 (Figure 1).

The list of potential farms with a population of 100 to 300 head of cattle was provided by the regional veterinary services. The cattle were managed under a stable-pasture system. From this list, only farms where the owners agreed to participate were included in the study. Sampling was carried out exclusively when the farm owner was present and gave informed consent. All cattle on these participating farms were eligible for examination and tick collection.

Each animal was carefully examined by visual inspection and palpation. Detected ticks were gently removed from the animal’s body using surgical forceps. All ticks were placed into plastic vials and transported to the laboratory for further examination at 4 °C. All ticks collected from a single animal were pooled into one tube. Ticks were identified morphologically using the tick identification keys [30,31,32,33] and a SZX12 trinocular stereomicroscope.

### 2.3. DNA Extraction from Ticks

Prior to DNA extraction, ticks were washed three times with physiological saline, followed by drying on filter paper at room temperature. Homogenization was performed in sterile tubes with Qiagen stainless steel beads using a TissueLyser LT homogenizer (Qiagen, Hilden, Germany) in 500 μL of lysis buffer from the “DNA-sorb-V” kit (AmpliSens, Moscow, Russia). After 5 min of homogenization, samples were incubated at 60 °C for 10 min and the homogenization procedure was repeated. This step was performed twice for each sample. Following homogenization and lysis, samples were centrifuged for 5 min at 3000× *g*, the liquid phase was transferred to a new tube, and extraction was completed according to the manufacturer’s protocol. DNA concentration was measured using a NanoDrop spectrophotometer (Thermo Fisher Scientific, USA).

### 2.4. Molecular Identification of Ixodid Ticks Using COX1 Primers

To confirm morphological identification of ticks to species level, conventional PCR followed by Sanger sequencing was performed for 113 ticks randomly selected from each collected tick species, ensuring equal representation of sexes, using primers targeting the *cox1* gene fragment (cytochrome c oxidase subunit 1). Samples were amplified by PCR on a GeneAmp PCR System 9700 (Applied Biosystems, Thermo Fisher Scientific, USA) using primers Cox1F 5′-GGAACAATATATTTAATTTTTGG-3′ and Cox1R 5′-ATCTATCCCTACTGTAAATATATG-3′ [34]. The 30 μL reaction mixture contained: 13.6 μL water, 3 μL 25 mM KCl, 3 μL 2 mM dNTP, 3 μL 25 mM MgCl_2_, 10 pmol of each primer, 1 U Taq DNA Polymerase (Syntol, Moscow, Russia), and 5 μL template DNA. PCR conditions consisted of: initial denaturation at 95 °C for 5 min; 30 cycles of 95 °C for 30 s, 54 °C for 60 s, and 72 °C for 60 s; final extension at 72 °C for 5 min. Amplification products were separated on a 1.5% agarose gel with ethidium bromide staining and visualized using a Gel Doc XR+ system (Bio-Rad, San Francisco, CA, USA). Expected fragment size was 800–820 bp.

### 2.5. PCR Amplification for the Detection of Theileria annulata in Ticks

PCR was performed using primers Eno_T.anul_F 5′-TTGCGAGATGGAGACAAAAGC-3′ and Eno_T.anul_R 5′-TCAGGGTGTGATAAACTTCTGCC-3′ targeting the *Enolase* gene [35]. The 25 μL reaction mixture per tube contained: 12.5 μL of ready-made BioMaster HS-Taq PCR-Spec mix (2×), 10 pmol of each primer, 5 μL of DNA, and deionized water up to a total reaction volume of 25 μL. The PCR amplification program included: initial denaturation at 95 °C for 5 min; 35 cycles of 95 °C for 30 s, 60 °C for 40 s, and 72 °C for 50 s; final extension at 72 °C for 5 min. Previously identified DNA samples, confirmed as positive for *Theileria* by sequencing, were used as positive controls. PCR was performed using a GeneAmp PCR System 9700 (Applied Biosystems, Thermo Fisher Scientific, Waltham, MA, USA). Expected fragment size was 451 bp.

### 2.6. PCR Amplification for the Detection of Anaplasma spp. in Ticks

PCR was performed using forward primer groEL_Anapl_all_F 5′-AAGGATGGATAYAAGGTMATGAA-3′ and reverse primer groEL_Anapl_all_R 5′-CGCGGWCAAACTGCATAC-3′ under amplification conditions described previously. DNA samples that had been confirmed as positive for *Anaplasma* by sequencing were used as positive controls [36].

### 2.7. Sequence Alignments and Phylogenetic Analyses

Amplified fragments from positive samples were sequenced using the Sanger method on a 3730xl DNA Analyzer (Applied Biosystems, Thermo Fisher Scientific, USA) with the BigDye^®^ Terminator v3.1 Cycle Sequencing Kit (Applied Biosystems, Thermo Fisher Scientific, USA), following the manufacturer’s protocol. Forward and reverse primer sequences were assembled and trimmed using SeqMan software (DNASTAR-Lasergene v6) and identified via the GenBank database using the BLAST algorithm [37]. For each phylogenetic tree, the nucleotide substitution model was selected individually based on the Bayesian Information Criterion (BIC) in MEGA v.12 [38]. Tree construction was performed using the Maximum Likelihood method with the corresponding selected model. Branch support was assessed by bootstrap analysis with 1000 replications. Bootstrap values ≥ 50% are shown on the trees. No outgroup was used for tree rooting. All alignment positions were included in the analysis, including the 1st, 2nd, and 3rd codon positions, as well as noncoding regions. Novel sequences obtained in the present study are indicated on the phylogenetic tree by a black triangle (▲) preceding the sequence name; GenBank accession numbers are provided for all sequences, including the new ones. Percentage identity between sequences was calculated using the MegAlign module of the Lasergene 6.0 software package (DNASTAR, Madison, WI, USA).

### 2.8. DNA Accession Numbers

The sequence data of ticks generated in this study were deposited in GenBank [39]. The accession numbers for the *cox1* gene fragments obtained in this study are as follows: PV810189 for *Dermacentor niveus*; PV810469 and PV810471 for *Rhipicephalus annulatus*; PV810709 and PV810710 for *Hyalomma asiaticum*; PV810384 and PV810385 for *Hyalomma marginatum*; PV810669-PV810701 for *Hyalomma scupense*; PX244311- PX244318 for *Theileria annulata* (Appendix A, Table A1).

### 2.9. Statistical Analysis

The tick infestation rate in animals was assessed using the occurrence index (OI) and the abundance index (AI).

OI = (Number of infested animals × 100)/Total number of examined animals;

AI = Total number of ticks/Total number of examined animals.

The Chi-square test of independence and Fisher’s exact test were used to establish associations between different categorical variables. The influence of potential risk factors (such as oblast, month, and tick species) on the likelihood of tick infestation was assessed by calculating odds ratios (OR) with corresponding 95% confidence intervals (CI). Statistical analyses were performed using EpiInfo 7 software (CDC, Atlanta, GA, USA). Statistical significance was set at *p* < 0.05.

## 3. Results

### 3.1. Prevalence and Species Composition of Ticks on Cattle

A total of 2499 cattle were examined, of which 738 animals (29.5%) were found to be infested with ixodid ticks (Table 1). In total, 3121 ticks were collected from infested animals, including 643 specimens (20.6%; 95% CI: 19.2–22.1%) from the Almaty oblast, 1168 specimens (37.4%; 95% CI: 35.7–39.1%) from the Zhambyl oblast, and 1310 specimens (42.0%; 95% CI: 40.3–43.7%) from the Turkistan oblast. Since all ticks were collected from cattle, they were all engorged with blood to varying extents.

**Figure 2:**
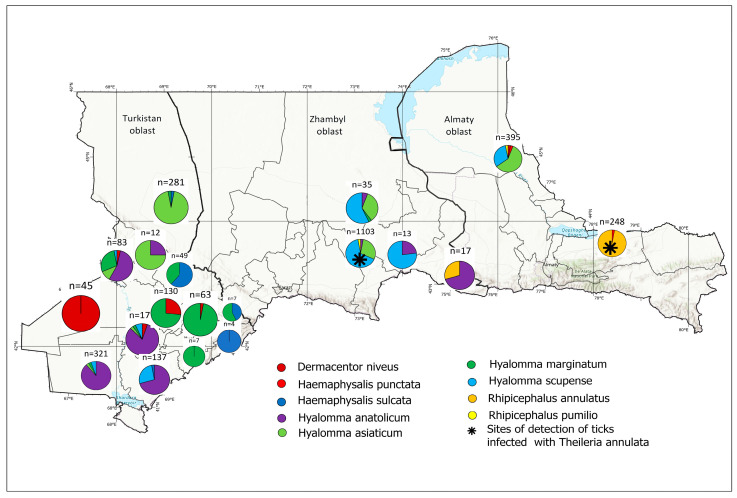
Sites of detection of ticks infected with *Theileria annulata*.

Morphological identification revealed the presence of nine species belonging to four genera (Table 1 and Figure 2). The most abundant genus was *Hyalomma*, with 2711 specimens (86.9%), followed by *Rhipicephalus* (266; 8.5%), *Dermacentor* (101; 3.2%), and *Haemaphysalis* (43; 1.4%). Among the *Hyalomma* species, *Hy. scupense* (31.7%; 990/3121), *Hy. asiaticum* (27.9%; 871/3121), *Hy. anatolicum* (19.6%; 611/3121), and *Hy. marginatum* (7.7%; 239/3121) were predominant. *Rh. annulatus* accounted for 8.1% (253/3121). Less frequently recorded species included *Ha. punctata* (0.2%; 7/3121), *Ha. sulcata* (1.2%; 36/3121), *Rh. pumilio* (0.4%; 13/3121), and *D. niveus* (3.2%; 101/3121).

**Figure 3:**
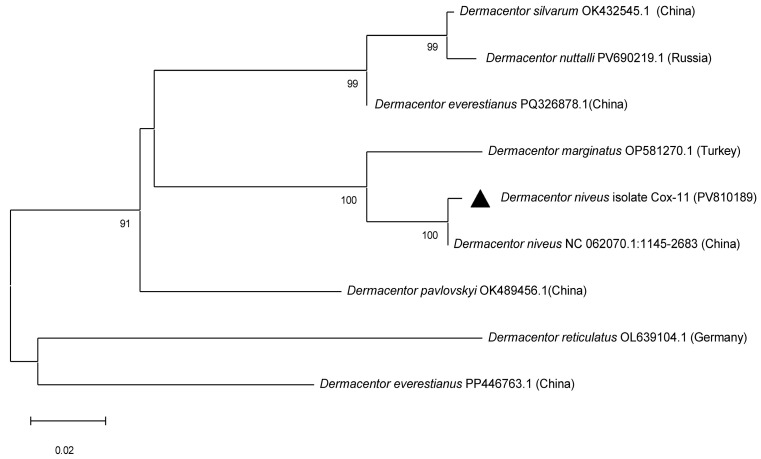
Phylogenetic tree constructed using the Maximum Likelihood method based on partial nucleotide sequences of the mitochondrial *cox1* gene for the identification of *Dermacentor* ticks. The Tamura three-parameter substitution model (T92) with a gamma-distributed rate variation among sites (+G) was applied. Branch support values were assessed by bootstrap analysis with 1000 replicates.

**Figure 4:**
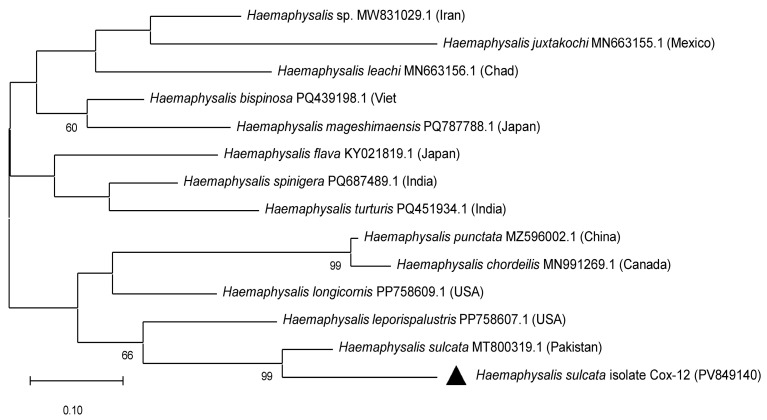
Phylogenetic tree constructed using the Maximum Likelihood method for the identification of *Haemaphysalis* ticks based on partial nucleotide sequences of the mitochondrial *cox1* gene. The Tamura–Nei 1993 substitution model (TN93) was applied. Branch support values were assessed by bootstrap analysis with 1000 replicates.

**Figure 5:**
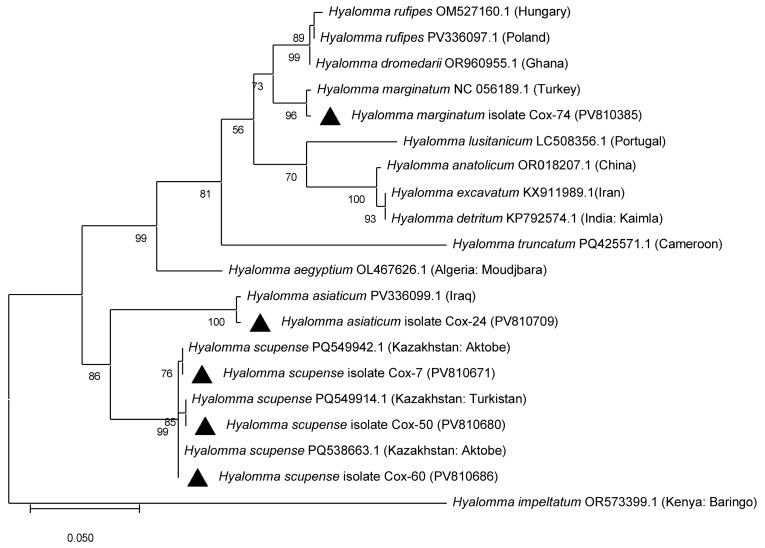
Phylogenetic tree constructed using the Maximum Likelihood method for the identification of *Hyalomma* ticks based on partial nucleotide sequences of the mitochondrial *cox1* gene. The Hasegawa–Kishino–Yano substitution model (HKY) was applied. Branch support values were assessed by bootstrap analysis with 1000 replicates.

**Figure 6:**
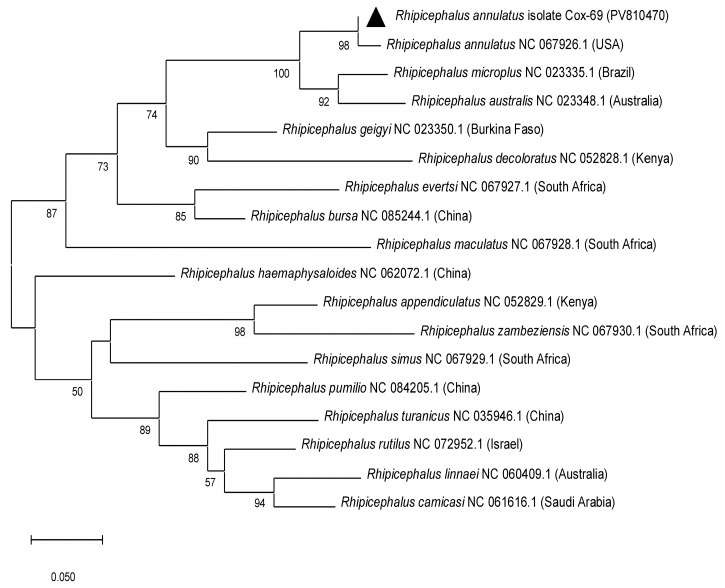
Phylogenetic tree constructed using the Maximum Likelihood method for the identification of *Rhipicephalus* ticks based on partial nucleotide sequences of the mitochondrial *cox1* gene. The General Time Reversible substitution model (GTR) was applied. Branch support values were assessed by bootstrap analysis with 1000 replicates.

PCR amplification of the mitochondrial *cox1* gene was successful in 55 out of 113 tick DNA samples. Sequencing of these amplicons enabled accurate molecular identification of tick species: 33 *Hyalomma scupense*, 2 *Hy. marginatum*, 2 *Hy. asiaticum*, 1 *Haemaphysalis sulcata*, 3 *Rhipicephalus annulatus*, and 1 *Dermacentor niveus* (Figure 3, Table A1). The obtained sequences showed high nucleotide identity (97.83–100%) with reference sequences of the corresponding tick species available in GenBank from China, Turkey, Iran, France, and the USA (Figure 3, Figure 4, Figure 5 and Figure 6, Table A1).

### 3.2. Analysis of Tick Infestation by Season, Region, and Species

**Figure 7:**
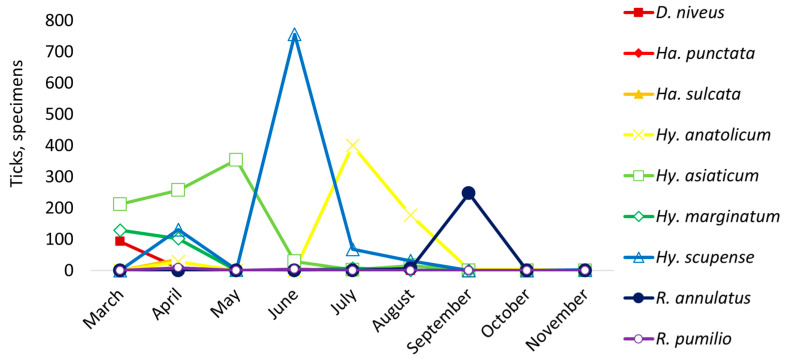
The number of ticks removed from cattle during the study period in the southern regions of Kazakhstan.

The main period of tick activity was observed in spring and early summer (Figure 7).

Tick infestation frequency showed significant variation across the months (χ^2^ = 1242.8, *p* < 0.001). Among all identified tick species, *Hy. scupense* was the most widespread, recorded in all three regions with peak activity in June (76.4%; 756/990; 95% CI: 73.6–78.9%), being virtually absent in early spring months. *Hy. scupense* was significantly more frequently detected on cattle in June compared to all other collection months (*p* < 0.0001), representing the predominant tick species among all those removed during that period.

*Hyalomma asiaticum* showed predominantly spring activity, with the highest prevalence in May (40.6%; 354/871; 95% CI: 37.4–44.0%) and a significant presence in March (26.1%) and April (29.5%), but its numbers sharply declined by summer. *Hy. anatolicum* was active later in the season, peaking in July (65.5%; 400/611; 95% CI: 61.6–69.1%), with high prevalence in August (30%), classifying it as a summer-active species.

Early spring activity was demonstrated by *Hy. marginatum*, peaking in March (54.0%; 129/239; 95% CI: 47.6–60.2%), while *D. niveus* was active exclusively in March (92.1%; 93/101; 95% CI: 85.1–95.9%) and April (7.9%; 8/101). *Rh. annulatus* peak activity was in October (97.6%; 247/253; 95% CI: 94.9–98.9%), *Rh. pumilio* was detected in April (0.47%, 9/13; 95% CI: 42.4–87.3%) and in June (30.8%, 4/13; 95% CI: 12.7–57.6%). Thus, the data suggests seasonal specialization of species: spring dominance by *Hy. asiaticum*, *Hy. marginatum* and *D. niveus*, early summer dominance by *Hy. scupense*, mid-summer dominance by *Hy. anatolicum*, autumn dominance by *Rh. annulatus*.

The highest cattle infestation frequency was observed in Zhambyl oblast (27.6%; 475/1723; OR = 1.7; 95% CI: 1.3–2.1; *p* < 0.0001) compared to Turkistan oblast (18.7%; 133/713; 95% CI: 16.0–21.7%). However, differences between Almaty and Zhambyl oblasts, as well as between Almaty and Turkistan oblasts, were not statistically significant. In Zhambyl oblast, the majority of specimens belonged to *Hy. scupense* (34.6%; 1076/1335) and *Hy. asiaticum* (28.9%; 560/1114). *Hy. anatolicum* (17.7%; 656/684) and *Hy. marginatum* (99.3%; 288/290) were predominantly found in Turkistan oblast, being completely absent in Almaty oblast. Similar localization was shown by *D. niveus* (92.0%; 103/112) and *Ha. sulcata* (100%; 41/41) which occurred exclusively in Turkistan oblast. In Almaty oblast, *Rh. annulatus* dominated (96.5%; 249/258), with *Rh. pumilio* and *Ha. punctata* also detected (10 specimens each). The χ^2^-test results (χ^2^ = 3608.32; df = 16; *p* < 0.0001) confirmed statistically significant differences in species distribution across regions.

### 3.3. Detection of Anaplasma spp. and Theileria annulata in Ticks

A total of 8 out of 113 samples (7.1%; 95% CI: 3.6–13.4%) tested positive for *Theileria annulata* by PCR (Table 2).

*Theileria annulata* was detected in 7 *Hy. scupense* nymphs (9.1%; 7/77; 95% CI: 4.5–17.6%) in late March and 1 adult female *Rh. annulatus* (11.1%; 1/9; 95% CI: 2.0–43.5%) in early September. All *Hy. scupense* positive ticks were collected in Zhambyl oblast, while *Rh. annulatus* was found in Almaty oblast. No *Anaplasma* spp. DNA was detected in the examined ticks.

## 4. Discussion

Tick-borne diseases of cattle, including theileriosis, pose a serious threat to livestock health and productivity worldwide, as they can cause substantial economic losses due to reduced milk and meat production, increased veterinary costs, and animal mortality [40]. Despite the global significance of these diseases, the epidemiology of tick infestations and the pathogens they transmit remains poorly understood in many regions, including Kazakhstan. To elucidate the mechanisms of pathogen transmission and provide a scientific basis for the development of region-specific control programs for parasitic infections, it is essential to investigate their taxonomic diversity, seasonal activity patterns, and epidemiological importance. In southern Kazakhstan, 18 species of ixodid ticks have been recorded [12,41,42], of which at least 12 species are known to parasitize cattle [13,43,44].

In the present study, nine tick species parasitizing cattle in the Turkistan, Zhambyl, and Almaty oblasts were identified: *Hy. anatolicum*, *Hy. asiaticum*, *Hy. scupense*, *Hy. marginatum*, *D. niveus*, *Rh. annulatus*, *Rh. pumilio*, *Ha. sulcata*, and *Ha. punctata*. Phylogenetic analysis based on the mitochondrial *cox1* gene revealed low genetic diversity among tick populations from Kazakhstan and high similarity with geographically distant isolates from Europe, Asia, and even America. This likely reflects the conservative nature of the mitochondrial *cox1* locus, which is widely used for species identification but has limited resolution for intraspecific genotyping and detection of fine-scale population structure. Therefore, to gain a more comprehensive understanding of the genetic diversity, population dynamics, and phylogeography of tick species in Kazakhstan, future studies should incorporate additional mitochondrial or nuclear markers, such as 16S rRNA, ITS2, or genome-wide SNP analysis.

**Figure 8:**
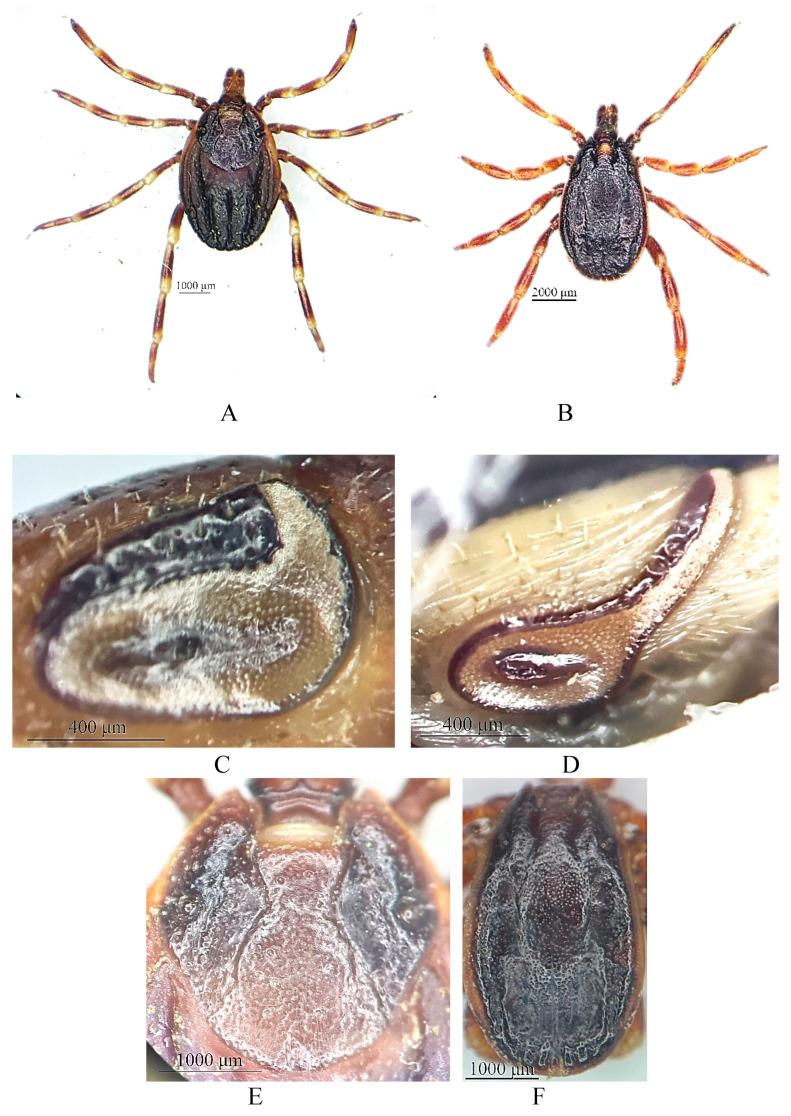
Picture of *Hyalomma marginatum* collected from cattle in Turkistan oblast: General view of female (**A**) and male (**B**); peritreme of female (**C**) and male (**D**); dorsal shield of female (**E**) and male (**F**).

The most dominant genus identified in this study was *Hyalomma*, consistent with previous reports indicating its prevalence in the arid and semi-arid regions of Central Asia [4,5,12,45,46]. This genus is well adapted to pastoral systems in the region and plays a significant role in the transmission of tick-borne pathogens [47], including *Theileria* spp. Among *Hyalomma* species in southern Kazakhstan, *Hy. scupense* and *Hy. anatolicum* are the most widely distributed. In the southern regions of Kazakhstan, these two species have adapted to habitats within and around human settlements and are most frequently encountered on cattle in semi-desert steppe and foothill zones. However, they have not been recorded in open desert landscapes. In Kazakhstan, *Theileria orientalis* and *T. equi* have been detected in *Hy. scupense* collected from horses in the Zhetysu oblast, whereas *T. equi* and *Anaplasma phagocytophilum* have been found in specimens from the Kyzylorda oblast. In addition, *T. annulata* and *Ehrlichia* sp. have been detected in ticks collected from cattle in the Kazygurt district of the Turkistan oblast [29]. *Hyalomma asiaticum* is more evenly distributed across desert areas, but occurs less frequently than *Hy. scupense* and *Hy. anatolicum*, reflecting its more limited ecological adaptability. *Hyalomma marginatum* (Koch, 1844) was initially identified based on morphological characteristics as *Hyalomma turanicum* (Pomerancev, 1946), since the examined specimens exhibited frequent small punctations and a narrow peritremal extension (Figure 8), features that distinguish them from the typical *Hy. marginatum*, which is characterized by sparse punctations and a broad extension.

Previously, the distribution ranges of these species were considered non-overlapping: *Hyalomma turanicum* in Kazakhstan was reported exclusively in the foothills of the western Tien Shan [8,12], whereas *Hy. marginatum* was recorded in the West Kazakhstan, Atyrau, and the western part of the Aktobe oblasts [48,49,50,51].

However, the results of the molecular genetic analysis conducted in the present study demonstrated complete nucleotide identity of the samples with *Hy. marginatum*, allowing their definitive assignment to this species. These findings are of particular importance for refining the distribution range of *Hy. marginatum* in Kazakhstan, revising taxonomic diagnostic criteria, and informing strategies for monitoring and controlling vector populations of transmissible diseases.

Ticks of the genera *Rhipicephalus*, *Dermacentor*, and *Haemaphysalis* were less frequently encountered, which can be attributed to their localized distribution in Kazakhstan [13,52]. The detection of *Rhipicephalus annulatus* in the Korday district of the Zhambyl oblast and the Enbekshikazakh district of the Almaty oblast indicates a substantial expansion of its range, likely associated with an increase in mean annual temperature [53]. Members of the genus *Rhipicephalus* are known vectors of theileriosis, anaplasmosis, and other tick-borne infections [54,55].

It is important to note that occasional findings of certain tick species in atypical areas are most often associated with insufficient veterinary control over transported or herded livestock, which facilitates the expansion of the range of foci of the diseases they transmit. Thus, *Hyalomma asiaticum* is a typical representative of desert fauna and a vector of numerous pathogens, including *Theileria* spp. [40] and *Anaplasma* spp. [56]—was most frequently detected on cattle in the desert zones of the Sozak district (Turkistan oblast), the Merke district (Zhambyl oblast), and the Balkhash district (Almaty oblast). Isolated specimens were also recorded on cattle in settlements of the Arys and Shardara districts. Notably, isolated findings of *Ha. sulcata* in the atypical desert zone of the Sozak district are, in our opinion, associated with accidental introduction resulting from livestock movement. *Ha. sulcata* is an important epizootiological vector, particularly in areas with developed animal husbandry [57].

The seasonal distribution of ticks observed in this study corresponded to well-established patterns of tick activity in temperate and semi-arid climates. Tick infestations were most pronounced in late spring and early summer, with a peak in June, which is characteristic of *Hyalomma* spp., as they are known to exhibit high activity during warm periods. The decline in infestation levels from August coincided with reduced tick activity during cooler months. Interestingly, in some regions, particularly in the Almaty oblast, *Rh. annulatus* displayed unusual late-season activity, suggesting potential local changes in the ecology of this species that merit further investigation.

Previous studies conducted in the Turkistan and Zhambyl oblasts have reported a high prevalence of bovine vector-borne parasitic diseases, including *Anaplasma phagocytophilum*, *A. ovis*, and *Theileria annulata* [25]. In the Kazygurt, Sairam, Saryagash, and Tulkibas districts, these pathogens were detected in *Hy. anatolicum*, *Hy. asiaticum*, *Hy. scupense*, and *Rh. turanicus*. In the Moiynkum district (Zhambyl oblast) and the Zhambyl district (Almaty oblast), *Hy. anatolicum* was identified as a vector of *T. annulata* [29].

In our study, molecular genetic analysis of ticks collected from cattle revealed the presence of *T. annulata* DNA in two species: *Hy. scupense* in the Zhambyl oblast (9.1%; 7/77) and, for the first time, in *Rh. annulatus* in the Almaty oblast (11.1%; 1/9). No *Anaplasma* spp. DNA was detected in any of the examined tick specimens. The absence of *Anaplasma* spp. in our tick samples suggests that this pathogen may not be widely distributed in the studied regions, a finding consistent with previous surveys of cattle for anaplasmosis [36].

These results underscore the importance of continuous monitoring of tick populations and the pathogens they transmit, as changes in tick species composition and pathogen prevalence can have significant implications for livestock health and agricultural productivity. Furthermore, the detection of *T. annulata* in ticks within the region highlights the need for targeted vector control strategies, including regular tick surveillance, the effective use of acaricides, and appropriate therapeutic measures for the treatment of theileriosis.

## 5. Conclusions

In the course of the study, nine species of ixodid ticks belonging to the genera *Hyalomma*, *Rhipicephalus*, *Dermacentor*, and *Haemaphysalis* were found parasitizing cattle. For the first time, *Rhipicephalus annulatus* was recorded in the Almaty and Turkistan oblasts.

Molecular genetic analysis did not detect the causative agent of bovine anaplasmosis (*Anaplasma marginale*) in the ticks examined during the observation period. However, *Theileria annulata* was identified in *Hyalomma scupense* (9.1%) in the Zhambyl oblast and in *Rh. annulatus* (11.1%) in the Almaty oblast. These findings confirm the well-established epidemiological role of *Hy. scupense* and, for the first time, indicate *Rh. annulatus* as a vector of *T. annulata* in this region.

The obtained data are of considerable importance for the prevention of theileriosis and for planning tick control measures, including the optimization of acaricidal treatment schedules for livestock.

## Figures and Tables

**Table 1:** Ticks collected from cattle in the Almaty, Zhambyl, and Turkistan oblasts of Kazakhstan in 2024.

Oblast	Number of Cattle Examined	Species	Number of Animals with Ticks (%)	Number of Ticks Collected (%)	OR (95% CI), *p*	Mean Number of Ticks per Animal
Almaty	63	*Hy. asiaticum*	9 (14.3)	242 (37.6)	0.02 (0.01–0.04), *p* < 0.0001	3.84
		*Hy. scupense*	3 (4.8)	130 (20.2)	0.04 (0.02–0.09), *p* < 0.0001	2.06
		*D. niveus*	1 (1.6)	8 (1.2)	0.9 (0.3–2.4), *p* = 0.8	0.13
		*Ha. punctata*	3 (4.8)	7 (1.1)	Ref. group	0.11
		*Rh. annulatus*	2 (3.2)	247 (38.4)	0.02 (0.01–0.04), *p* < 0.0001	3.92
		*Rh. pumilio*	1 (1.6)	9 (1.4)	0.8 (0.3–2.1), *p* = 0.6	0.14
Total in the oblast			19 (30.2)	643 (20.6)	Ref. group	0.21
Zhambyl	1723	*Hy. anatolicum*	8 (0.5)	20 (7 adults and 13 nymphs) (1.7)	0.05 (0.01–0.37), *p* = 0.003	0.01
		*Hy. asiaticum*	213 (12.4%)	347 (29.7)	0.002 (0.0003–0.02), *p* < 0.0001	0.20
		*Hy. scupense*	285 (16.5)	791 (756 adults and 35 nymphs) (67.7)	0.0004 (0.0001–0.003), *p* < 0.0001	0.46
		*Hy. marginatum*	1 (0.06)	1 (0.1)	Ref. group	0.001
		*Rh. annulatus*	2 (0.1)	5 (1 adult and 4 nymphs) (0.4)	0.2 (0.02–1.7), *p* = 0.1	0.003
		*Rh. pumilio*	1 (0.06)	4 (0.3)	0.3 (0.03–2.2), *p* = 0.2	0.002
Total in the oblast			510 (29.6)	1168 (37.4)	0.4 (0.4–0.5), *p* < 0.0001	0.68
Turkistan	713	*Hy. anatolicum*	65 (9.1)	591 (561 adults and 30 nymphs) (45.1)	0.001 (0.0001–0.01), *p* < 0.0001	0.83
		*Hy. asiaticum*	21 (3.0)	282 (21.5)	0.003 (0.0004–0.02), *p* < 0.0001	0.4
		*Hy. scupense*	57 (8.0)	69 (5.3)	0.01 (0.002–0.1), *p* < 0.0001	0.1
		*Hy. marginatum*	50 (7.0)	238 (18.2)	0.003 (0.0005–0.03), *p* < 0.0001	0.33
		*D. niveus*	10 (1.4)	93 (7.1)	0.01 (0.001–0.07), *p* < 0.0001	0.13
		*Ha. sulcata*	5 (0.7)	36 (2.8)	0.03 (0.004–0.2), *p* = 0.0004	0.05
		*Rh. annulatus*	1 (0.1)	1 (0.1)	Ref. group	0.001
Total in the oblast			209 (29.3)	1310 (42.0)	0.4 (0.3–0.4), *p* < 0.0001	1.84
TOTAL	2499		738 (29.5)	3121 (3039 adults and 82 nymphs)		1.25

**Table 2:** *Theileria annulata* in ticks collected from cattle in the Almaty, Zhambyl, and Turkistan oblasts of Kazakhstan in 2024, according to tick species.

Family	Genus	Species	Number of Examined	Number of Positive (%)	95% CI	Number of Examined	Number of Positive (%)	OR (95% CI), *p*
Ixodidae	*Dermacentor*	*D. niveus*	4	0 (0.0)	-	4	0 (0.0)	-
	*Haemaphysalis*	*Ha. sulcata*	2	0 (0.0)	-	4	0 (0.0)	-
		*Ha. punctata*	2	0 (0.0)	-			
	*Hyalomma*	*Hy. anatolicum*	6	0 (0.0)	-	94	7 (7.5)	1.2 (0.1–11.2); 0.8
		*Hy. asiaticum*	9	0 (0.0)	-			
		*Hy. scupense*	77	7 (9.1)	4.5–17.6			
		*Hy. marginatum*	2	0 (0.0)	-			
	*Rhipicephalus*	*Rh. annulatus*	9	1 (11.1)	2.0–43.5	11	1 (9.1)	Ref. group
		*Rh. pumilio*	2	0 (0.0)	-			
TOTAL			113	8 (7.1)	3.6–13.4			

## Data Availability

The original contributions presented in this study are included in the article. Further inquiries can be directed to the corresponding author.

## Appendix A

**Table A1:** Nucleotide sequences of the *cox1* gene locus generated using Sanger sequencing of *Hy. scupense* (n = 33), *Hy. marginatum* (n = 2), *Hy. asiaticum* (n = 2), *Ha. sulcata* (n = 1), *D. niveus* (n = 1), and *Rh. annulatus* (n = 3) collected in Almaty, Zhambyl and Turkistan oblasts of Kazakhstan.

#	GenBank ID	Tick Species, Oblast	% of Identity with the Closest Tick Species (GenBank ID (Country of Detection))
1	PV810669	*Hy. scupense*, Zhambyl	100.00% (NC_062166.1, China)
2	PV810670	*Hy. scupense*, Turkistan	99.42% (KX000638.1, France)
3	PV810671	*Hy. scupense* Turkistan	99.26% (KX000638.1, France)
4	PV810672	*Hy. scupense* Turkistan	99.03% (KX000638.1, France)
5	PV810673	*Hy. scupense*, Turkistan	99.34% (NC_062166.1, China)
6	PV810674	*Hy. scupense*, Turkistan	99.84% (KX000638.1, France)
7	PV810675	*Hy. scupense*, Turkistan	99.83% (KX000638.1, France)
8	PV810676	*Hy. scupense*, Turkistan	99.86% (NC_062166.1, China)
9	PV810677	*Hy. scupense*, Turkistan	99.31% (KX000638.1, France)
10	PV810678	*Hy. scupense*, Turkistan	99.02% (KX000638.1, France)
11	PV810679	*Hy. scupense*, Zhambyl	99.87% (NC_062166.1, China)
12	PV810680	*Hy. scupense*, Zhambyl	100.00% (NC_062166.1, China)
13	PV810681	*Hy. scupense*, Zhambyl	99.55% (NC_062166.1, China)
14	PV810682	*Hy. scupense*, Zhambyl	98.92% (MW546282.1, Turkey)
15	PV810683	*Hy. scupense*, Zhambyl	99.74% (NC_062166.1, China)
16	PV810684	*Hy. scupense*, Zhambyl	99.61% (NC_062166.1, China)
17	PV810685	*Hy. scupense*, Zhambyl	99.73% (NC_062166.1, China)
18	PV810686	*Hy. scupense*, Zhambyl	99.44% (KX000638.1, France)
19	PV810687	*Hy. scupense*, Zhambyl	100.00% (KF583581.1, China)
20	PV810688	*Hy. scupense*, Zhambyl	100.00% (NC_062166.1, China)
21	PV810689	*Hy. scupense*, Turkistan	99.32% (MW546282.1, Turkey)
22	PV810690	*Hy. scupense*, Turkistan	99.17% (MW546282.1, Turkey)
23	PV810691	*Hy. scupense*, Zhambyl	99.71% (NC_062166.1, China)
24	PV810692	*Hy. scupense*, Zhambyl	99.84% (NC_062166.1, China)
25	PV810693	*Hy. scupense*, Zhambyl	98.57% (NC_062166.1, China)
26	PV810694	*Hy. scupense*, Zhambyl	99.87% (NC_062166.1, China)
27	PV810695	*Hy. scupense*, Zhambyl	99.81% (MW546282.1, Turkey)
28	PV810696	*Hy. scupense*, Zhambyl	100.00% (MW546282.1, Turkey)
29	PV810697	*Hy. scupense*, Zhambyl	99.71% (NC_062166.1, China)
30	PV810698	*Hy. scupense*, Zhambyl	99.59% (NC_062166.1, China)
31	PV810699	*Hy. scupense*, Zhambyl	99.71% (MW546282.1, Turkey)
32	PV810700	*Hy. scupense*, Zhambyl	97.83% (NC_062166.1, China)
33	PV810701	*Hy. scupense*, Zhambyl	99.86% (NC_062166.1, China)
34	PV810384	*Hy. marginatum*, Turkistan	100.00% (MN885800.1, Turkey)
35	PV810385	*Hy. marginatum*, Zhambyl	99.71% (KX000644.1, China)
36	PV810709	*Hy. asiaticum*, Zhambyl	99.86% (KU364317.1, Kazakhstan)
37	PV810710	*Hy. asiaticum*, Zhambyl	100.00% (OQ415439.1, China)
38	PV810189	*D. niveus*, Almaty	99.74% (NC_062070.1, China)
39	PV849140	*Ha. sulcata*, Turkistan	99.50% (MH532299.1, Iran)
40	PV810469	*Rh. annulatus*, Almaty	99.54% (KX228542.1, USA)
41	PV810470	*Rh. annulatus*, Almaty	98.79% (KX228542.1, USA)
42	PV810471	*Rh. annulatus*, Zhambyl	99.39% (KX228542.1, USA)
43	PX244311	*SUB15577952 Thel_50*	100.00% (JN696671.1, China)
44	PX244312	*SUB15577952 Thel_51*	100.00% (JN696671.1, China)
45	PX244313	*SUB15577952 Thel_52*	100.00% (JN696678.1, China)
46	PX244314	*SUB15577952 Thel_53*	100.00% (JN696671.1, China)
47	PX244315	*SUB15577952 Thel_54*	100.00% (JN696671.1, China)
48	PX244316	*SUB15577952 Thel_55*	100.00% (JN696671.1, China)
49	PX244317	*SUB15577952 Thel_56*	100.00% (JN696671.1, China)
50	PX244318	*SUB15577952 Thel_65*	100.00% (JN696671.1, China)
